# Inner ear cell therapy targeting hereditary deafness by activation of stem cell homing factors

**DOI:** 10.3389/fphar.2015.00002

**Published:** 2015-01-27

**Authors:** Kazusaku Kamiya

**Affiliations:** Department of Otorhinolaryngology, Faculty of Medicine, Juntendo University, Tokyo, Japan

**Keywords:** hereditary deafness, stem cell homing, mesenchymal stem cell, inner ear cell therapy, connexin26

## Abstract

Congenital deafness affects about 1 in 1000 children and more than half of them have a genetic background such as Connexin26 (CX26) gene mutation. Inner ear cell therapy for sensorineural hearing loss has been expected to be an effective therapy for hereditary deafness. Previously, we developed a novel strategy for inner ear cell therapy using bone marrow mesenchymal stem cells as a supplement for cochlear fibrocytes functioning for cochlear ion transport. For cell therapy targeting hereditary deafness, a more effective cell delivery system to induce the stem cells into cochlear tissue is required, because gene mutations affect all cochlear cells cochlear cells expressing genes such as *GJB2* encoding CX26. Stem cell homing is one of the crucial mechanisms to be activated for efficient cell delivery to the cochlear tissue. In our study, monocyte chemotactic protein-1, stromal cell-derived factor-1 and their receptors were found to be a key regulator for stem cell recruitment to the cochlear tissue. Thus, the activation of stem cell homing may be an efficient strategy for hearing recovery in hereditary deafness.

## HEREDITARY DEAFNESS AND THE MOUSE MODELS ASSOCIATED WITH IONIC HOMEOSTASIS IN COCHLEA

Approximately one in 1,000 children is affected by severe hearing loss at birth or during early childhood, which is defined as pre-lingual deafness (Morton, 1991; Petersen and Willems, 2006) with about half of the cases attributable to genetic causes (Birkenhager et al., 2010). Among >100 known forms of non-syndromic deafness with identified genetic loci, by far the most common and best characterized is the one associated with *GJB2* (OMIM 121011), the gene encoding the connexin26 (CX26) protein (Kelsell et al., 1997). Recently we demonstrated that a mutation in CX26 induces macromolecular degradation of large gap junction plaque (GJP) complexes by using a CX26 dominant negative transgenic (Inoshita et al., 2014) and deficient mouse model (Kamiya et al., 2014). This molecular pathogenesis called “GJP disruption” is thought to be associated with a decreased endocochlear potential (EP) generated by cochlear ion transport. GJP disruption was also found in a mouse model for deafness type 3 (DFN3), which is the most common type of in X chromosome–linked, non-syndromic hearing loss. This mouse model has a deficiency of transcription factor Brn4 encoded by POU domain, class 3, transcription factor 4 (POU3F4; Kidokoro et al., 2014). We reported that the low EP observed in this mouse appeared to be caused by GJP disruption. A mouse model with a mutation in BSND, which encodes barttin, shows Bartter syndrome type IV, a hearing impairment (Nomura et al., 2013) also thought to be caused by a low EP.

## INNER EAR CELL THERAPY TARGETING COCHLEAR FIBROCYTE

Previously, we demonstrated that local inflammation at the lateral wall promotes the invasion of transplanted mesenchymal stem cells (MSCs) to the lateral wall (Kamiya et al., 2007). In our microarray analysis with cochlear mRNA after 3-nitropropionic acid (3-NP) administration, we showed the up-regulation of chemokine MCP-1 (monocyte chemotactic protein-1) which is known to be a stem cell homing factor (Belema-Bedada et al., 2008). Thus, the invasion of MSCs shown in our previous study was thought to be enhanced by stem cell homing factors.

## STEM CELL HOMING

Stem cell homing is one the crucial mechanisms to be activated for efficient cell delivery to the cochlear tissue. Belema-Bedada et al. (2008) demonstrated that efficient homing of multipotent adult MSCs into hearts damaged by ischemia/reperfusion was dependent on FROUNT-Mediated clustering of chemokine (C-C motif) receptor 2 (CCR2). They showed that the cytokine receptor CCR2 is necessary for organ-specific homing of pluripotent adult MSCs and that these cells are recruited by the CCR2 ligand MCP-1/CCL2 and express stromal cell-derived factor 1 (SDF-1), which might trap additional bone marrow-derived circulating cells to contribute to the complex process of homing and retention of circulating stem and progenitor cells to remodel diseased organs. As for homing to cochlea, Tan et al. (2008) demonstrated the homing capability of bone marrow-derived cells to a deafened cochlea. They suggested that the upregulation of SDF-1 in the spiral ligament after acoustic deafening promoted homing of the cell to the cochlea (Tan et al., 2008).

## ACTIVATION OF STEM CELL HOMING FACTORS IN COCHLEA AND STEM CELLS

**FIGURE 1:**
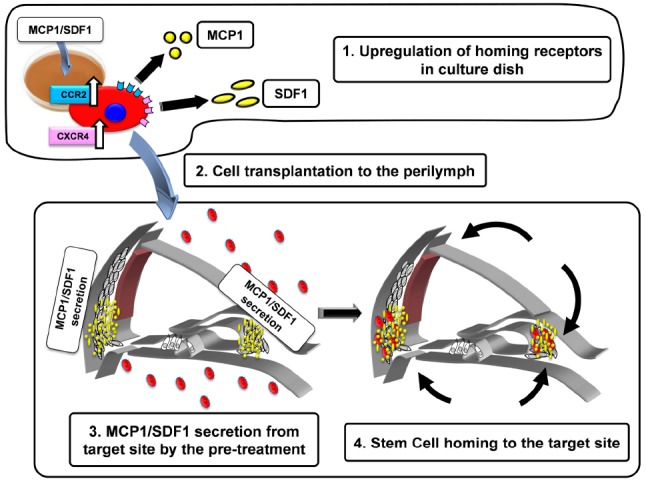
**Schematic diagram of the strategy to activate stem cell homing in the mouse cochlea.** 1. Upregulation of homing receptors (CCR2, CXCR4) in culture dish by the treatment of MCP-1 or SDF-1. The cells with abundant CCR2 and CXCR4 acquire the higher potentials to be attracted by MCP-1 and SDF-1 (black arrows). 2. The pretreated cells are injected into the perilymph via the semicircular canal. 3. MCP-1/SDF-1 secretion from the target site by pre-treatment with 3-nitropropionic acid (3-NP). 4. Transplanted cells home to the target site.

To establish an efficient cell therapy for hereditary deafness, we examined treatments that enhanced stem cell homing factors in the cochlear tissue and MSC. We examined inner ear MSC transplantation in CX26 deficient mice, which we developed as a model of hereditary hearing loss (Kamiya et al., 2014). We transplanted the MSCs to the lateral semicircular canal after the induction of stem cell homing factors (SDF-1; MCP-1) in the host cochlear tissue, and their receptors in transplanted MSCs. To enhance the invasion by MSCs of the cochlear tissue, we developed a novel transplant strategy by inducing SDF-1/MCP-1 expression in the host cochlear tissue and enhanced the expression of their receptors, CCR2 and C-X-C chemokine receptor type 4 (CXCR4) in MSCs (Figure 1). With this strategy, we induced efficient invasion of MSCs to the inner ear tissue and differentiation to form gap junctions with CX26 among transplanted MSCs in CX26-deficiente mouse inner ear (unpublished observation).

By using newly generated mouse models and a new strategy for the activation of stem cell homing factors, it may be possible to establish an efficient inner ear cell therapy for recovering hearing in hereditary deafness.

### Conflict of Interest Statement

The author declares that the research was conducted in the absence of any commercial or financial relationships that could be construed as a potential conflict of interest.
